# Boldine Attenuates Mechanical Allodynia and Mitigates Transcriptomic Changes in a Mouse Model of Chronic Constriction Injury

**DOI:** 10.3390/biomedicines14102345

**Published:** 2026-10-15

**Authors:** Jiangping Pan, Shahrukh Khalique, Alexey Kozlenkov, Carlos A. Toro, Christopher P. Cardozo, Wei Zhao

**Affiliations:** 1 Spinal Cord Damage Research Center, James J. Peters Veterans Affairs Medical Center, Bronx, NY 10468, USA; jiangping.pan@va.gov (J.P.); carlos.torochacon@va.gov (C.A.T.); christopher.cardozo@mssm.edu (C.P.C.); 2 VA Sequencing Collaborations United for Research and Epidemiology (seqCURE) Program, James J. Peters Veterans Affairs Medical Center, Bronx, NY 10468, USA; shahrukh.khalique@va.gov (S.K.);; 3 Department of Psychiatry, Icahn School of Medicine at Mount Sinai, New York, NY 10029, USA; 4 Department of Medicine, Icahn School of Medicine at Mount Sinai, New York, NY 10029, USA; 5 Department of Rehabilitation and Human Performance, Icahn School of Medicine at Mount Sinai, New York, NY 10029, USA

**Keywords:** neuropathic pain, mechanical allodynia, transcriptomic, nutraceutical

## Abstract

**Background/Objectives**: Neuropathic pain is a complex chronic disorder affecting roughly 7–10% of the population. Current neuropathic pain treatments fail to provide adequate relief in at least ~40% of patients and are usually accompanied by side effects that are sometimes severe. In this study, we investigated the effect of boldine, a natural occurring alkaloid, in attenuating peripheral nerve injury-induced neuropathic pain symptoms and transcriptomic changes. **Methods**: Male C57BL6 mice underwent chronic constriction injury (CCI) to the sciatic nerve. Customized diet containing boldine or control diet was administered immediately after surgery. Mechanical pain responses were measured by Von Frey filament test. Spontaneous pain-like weight bearing behavior was assessed using the dynamic weight bearing system. L3–L5 spinal cords were collected 15 days after injury and subjected to quantitative PCR and bulk RNA sequencing. **Results**: CCI led to ipsilateral hypersensitivity of mechanical pain responses and weight-bearing deficits including reduced rearing duration, ipsilateral/contralateral weight support ratio, and ipsilateral hind paw posed duration. Boldine treatment substantially attenuated CCI-induced mechanical allodynia but not the weight bearing deficits. Gene expression analysis showed that boldine inhibited CCI-induced upregulation of inflammatory mediators in the spinal cord. RNA sequencing analysis revealed several pain-related canonical pathways and upstream factors after CCI, and boldine treatment markedly reversed these CCI-induced transcriptomic changes. **Conclusions**: Early oral administration of boldine attenuated CCI-induced mechanical allodynia and was associated with reduced inflammatory gene expression and reversal of multiple injury-related transcriptomic changes in the spinal cord. These preliminary preclinical findings support further investigation of boldine and do not establish therapeutic efficacy in established neuropathic pain.

## 1. Introduction

Peripheral nerve injury (PNI)-induced neuropathic pain is a condition resulting from damage to the peripheral nervous system [1]. PNI is characterized by symptoms such as allodynia, hyperalgesia, and spontaneous pain. These injuries, often caused by trauma or surgery, disrupt normal sensory signaling, leading to dysfunctional neural plasticity in both peripheral and central nervous systems. Neuropathic pain typically correlates with diminished physical and psychological well-being, severely compromising a patient’s overall quality of life [2]. Current treatments of neuropathic pain include anticonvulsants, tricyclic antidepressants, serotonin inhibitors, and opioids, but they rarely provide complete resolution of symptoms and are associated with significant side effects [1,3]. Thus, there is a great need for developing new therapy with improved efficacy, tolerability and fewer side effects.

In recent years, non-pharmacological supplements and nutraceuticals are increasingly recognized for their potential as complementary therapies that may optimize the efficacy of conventional pharmaceutical treatments for chronic neuropathic pain. For example, palmitoylethanolamide has been shown to alleviate neuropathic pain through inhibiting inflammation and providing neuroprotection [4]. Curcumin mitigates pain behavior and reduces inflammatory markers including IL-1β and TNF-α in preclinical neuropathy models including alcoholic neuropathy [5], chemotherapy-induced peripheral neuropathy [6,7,8], and diabetic painful neuropathy [9,10,11]. Resveratrol reduces pain-related behaviors by re-establishing homeostasis between pro- and anti-inflammatory pathways in the central nervous system following peripheral nerve injury [12]. Boldine, an alkaloid naturally isolated from the Chilean boldo tree (*Peumus boldus*), has demonstrated potent antioxidant and anti-inflammatory properties across various in vivo models [13,14,15,16]. Accumulating evidence from our laboratory and independent research groups demonstrates the therapeutic efficacy of boldine across various neurological conditions including Alzheimer’s disease [17], nerve repair [18], spinal cord injury [19,20], and most recently in spared nerve injury (SNI)-induced neuropathic pain in female mice [21].

Peripheral nerve injury triggers significant transcriptomic changes in the spinal cord, particularly within the dorsal horn where sensory information is processed. These changes involve alterations in gene expression related to inflammation, pain signaling, neuronal plasticity, and glial cell activity [22]. In this study, we tested whether oral administration of boldine could attenuate neuropathic pain-related behavioral changes in a mouse model of chronic constriction injury (CCI) to the sciatic nerve and whether boldine could normalize CCI-induced transcriptomic changes in the spinal cord.

## 2. Materials and Methods

### 2.1. Animals, Surgery, and Boldine Administration

Eight- to ten-week-old male C57BL6 mice were purchased from the Jackson Laboratory (Bar Harbor, ME, USA) and pair housed. Animals were housed in a temperature-controlled (20 ± 2 °C) vivarium under a standard 12 h light/dark cycle, with free access to food and water. All experimental protocols were reviewed and approved by the Institutional Animal Care and Use Committee (IACUC) at James J. Peters Veterans Affairs Medical Center (Protocol number #1585874) in strict accordance with established guidelines for animal welfare. Chronic constriction injury (CCI) was induced under general anesthesia (3–5% isoflurane). Following a cutaneous incision, the left sciatic nerve was exposed by blunt dissection through the connective tissue planes separating the gluteus superficialis and biceps femoris muscles. Three chromic gut ligatures were loosely tied around the nerve trunk at 1 mm intervals. Care was taken to ensure the knots were securely snug without arresting epineural blood flow. The muscular layer was closed using sutures, and the skin incision was secured with surgical staples [23,24]. Sham-operated control animals underwent an identical surgical exposure of the left sciatic nerve without the placement of ligatures. Boldine (Sigma-Aldrich, St. Louis, MO, USA) was administered immediately after surgery through a customized chow (OpenStandard Diet With 15 kcal% Fat and 275 mg Boldine per Kg Diet, #D21012101, Research Diets, New Brunswick, NJ, USA) formulated to provide a target exposure of approximately 50 mg/kg body weight/day. Dietary administration was selected to provide sustained oral exposure while avoiding repeated handling and gavage during longitudinal behavioral testing. Food consumption was monitored weekly and used together with body weight to estimate average boldine exposure. Based on the observed food intake of approximately 2.5–3.0 g/mouse/day, estimated exposure was approximately 50–60 mg/kg body weight/day. Because animals were pair housed, these values represent estimated average exposure rather than precise individual daily dosing. A control diet (OpenStandard Diet With 15 Kcal% Fat, #D11112201, Research Diets) was provided to sham-operated and untreated CCI groups. Animals were sacrificed 15 days after surgery. L3–L5 spinal cord tissue was dissected and stored at −80 °C for further analysis. The L3–L5 region was selected to sample lumbar circuitry receiving hindlimb sensory input; however, it does not encompass the entire spinal representation associated with the sciatic nerve.

### 2.2. Von Frey Filament Test

To assess mechanical pain thresholds, the von Frey monofilament test was performed on both the ipsilateral (injured) and contralateral (uninjured) hind paws [25]. Briefly, von Frey monofilaments (Bioseb, Vitrolles, France) were applied perpendicularly from beneath an elevated wire mesh platform to the plantar surface of the hind paw, beginning with the lowest gauge filament. Graded pressure was applied slowly until the filament bent. The procedure was repeated using filaments of increasing bending forces until a nociceptive response such as paw flicking, lifting, licking, or vocalization was elicited. The withdrawal threshold was defined and recorded as the minimum filament force required to evoke a positive withdrawal response.

### 2.3. Weight-Bearing Test

Weight-bearing behavior was assessed using the Dynamic Weight Bearing (DWB) 2.0 system (Bioseb, Vitrolles, France) as previously described [21]. Briefly, each animal was placed inside the DWB chamber for a 5 min testing period. Integrated floor sensors automatically captured the weight distribution and contact surface area of the paws relative to other body parts throughout the 5 min session. Postural and weight-bearing data were subsequently processed using the manufacturer’s automated tracking software.

### 2.4. RNA Isolation, cDNA Synthesis, and Quantitative PCR

Frozen L3–L5 spinal cords were homogenized in TRI-Reagent (Sigma-Aldrich, St. Louis, MO, USA) using a FastPrep-24™ 5G Bead Beating Grinder and Lysis System (MP Biomedicals, Solon, OH, USA). Total RNA was isolated following the manufacturer’s instructions. RNA concentrations were measured using a NanoDrop Spectrophotometer (Thermo Fisher Scientific, Waltham, MA, USA), and 1 µg total RNA was reverse transcribed using High-Capacity cDNA Reverse Transcription Kit (Applied Biosystems, Carlsbad, CA, USA) followed by real time PCR using the TaqMan Universal PCR Master Mix on a ViiA 7 Real-Time PCR System (Applied Biosystems). TaqMan probes used were CCL2 (#Mm00441242_m1), NLRP3 (#Mm00840904_m1), IL-1β (#Mm00434228_m1), and TNF-α (#Mm00443258_m1, all from Applied Biosystems). Relative expression levels were quantified using the 2^−^^∆∆Ct^ method with hypoxanthine phosphoribosyltransferase 1 (HPRT) serving as the endogenous reference gene.

### 2.5. RNA Sequencing and Data Analysis

Library preparation and RNA sequencing (*N* = 4 per group) were performed by the VA Sequencing Collaborations United for Research and Epidemiology (seqCURE) Program at the James J. Peters VA Medical Center. Briefly, polyadenylated transcripts were selectively isolated from total RNA using poly-T oligo-conjugated magnetic beads. The enriched messenger RNA (mRNA) was thermally fragmented, followed by first-strand complementary DNA (cDNA) synthesis utilizing random hexamer primers and reverse transcriptase. Subsequently, second-strand synthesis was executed to yield double-stranded cDNA using the TruSeq RNA Library Prep Kit (Illumina, San Diego, CA, USA). Following sequential end repair, A-tailing, and adapter ligation, the resulting cDNA libraries were amplified via PCR and purified. Library quality, concentration, and fragment size distributions were verified on an Agilent 4200 TapeStation system (Agilent Technologies, Santa Clara, CA, USA). Validated libraries were then pooled and sequenced on an Illumina NextSeq 2000 platform to generate 2 × 75 bp paired-end reads with a sample-level sequencing depth of ~52 million ± 5 million. Raw sequencing reads were subjected to quality control using FastQC (v0.1.3) [26] (Post-QC and trimming read counts: ~20 million +/− 2 million). Low-quality bases and adapter sequences were trimmed using Trimmomatic (v0.39) [27], and reads failing minimum quality thresholds were discarded. High-quality reads were aligned to the Mus musculus reference genome (mm10, GRCm38) using the STAR aligner (v2.7.11b) [28] (STAR mapping rate: 90 ± 0.6% uniquely mapped reads). Gene-level read counts were generated using featureCounts (v2.26.0) [29]. To reduce noise from very low-abundance transcripts, genes with fewer than 15 mapped reads across all samples were excluded from downstream analysis. Normalization and differential expression analysis were performed using edgeR (v4.10.5) [30]. The RNA-seq analysis included four independent biological replicates per experimental group and was intended as an exploratory, hypothesis-generating analysis. For the exploratory analysis reported here, genes meeting nominal *p* < 0.05 and |logFC| > log2(1.2) were carried forward for pathway analysis. Because this nominal threshold does not control the false-discovery rate across the transcriptome, gene-level results are interpreted as exploratory and hypothesis-generating. Identified genes were subjected to pathway enrichment and network analysis using Ingenuity Pathway Analysis (IPA; Qiagen, Hilden, Germany).

### 2.6. Statistical Analysis

Statistical analyses were performed using GraphPad Prism 10, with data expressed as the mean ± standard error of the mean (SEM). To evaluate differences in mechanical withdrawal thresholds and weight-bearing parameters across experimental cohorts, a two-way repeated-measures analysis of variance (ANOVA) was executed, followed by a post-hoc multiple comparisons test. Relative gene expression profiles were compared among groups using a one-way ANOVA. For all statistical models, significance was predefined at *p* < 0.05.

## 3. Results

### 3.1. Boldine Attenuated CCI-Induced Mechanical Allodynia

**Figure 1:**
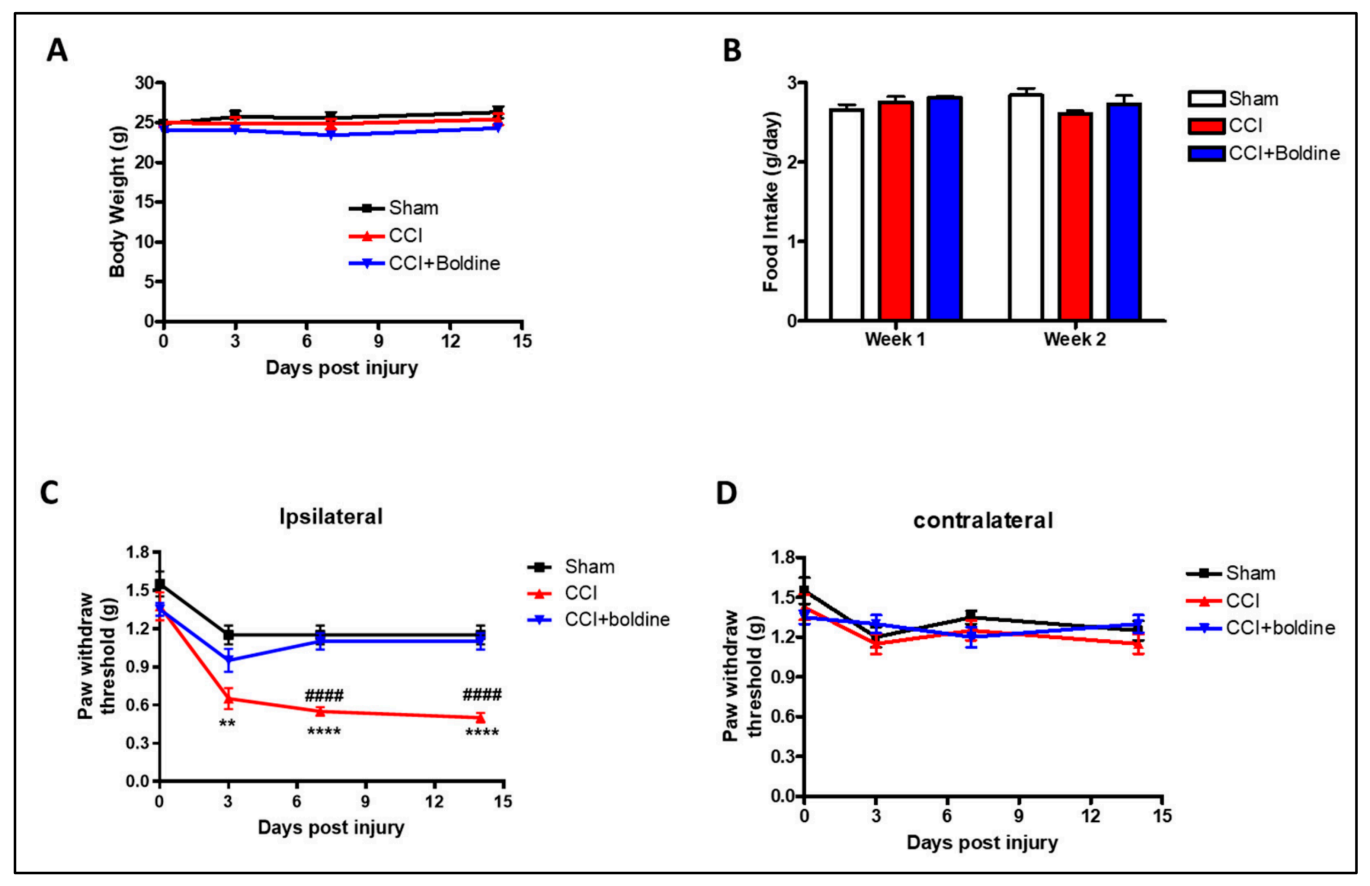
Boldine attenuated CCI-induced mechanical allodynia. Ten-week-old male C57BL6 mice underwent chronic constriction injury to the sciatic nerve. Boldine was orally administered immediately after injury via a customized diet. A control diet was provided to the sham-operated and CCI group. (**A**) Body weight was measured before surgery and at 3, 7 and 14 days post injury; (**B**) Average daily food intake per animal. (**C**) Withdraw threshold to mechanical stimuli on the ipsilateral hind paw as measured by von Frey filament test; (**D**) Withdraw threshold to mechanical stimuli on the contralateral hind paw. Data represent Mean ± SEM, N = 8 per group, ** *p* < 0.01, **** *p* < 0.0001 (CCI vs. sham), #### *p* < 0.001 (CCI + boldine vs. CCI) by two-way ANOVA with Tukey post-tests.

To evaluate the role of boldine in PNI-induced neuropathic pain, we conducted experiments in a mouse model of chronic constriction injury to the sciatic nerve, which is an established preclinical model of PNI [31]. Ten-week-old male C57BL6 mice underwent CCI or sham surgery. Customized diet containing boldine or control diet was administered immediately after surgery. Behavioral assessments, including von Frey testing and dynamic weight bearing, were performed before injury for baseline and beginning 3 days post injury. Food consumption was monitored weekly. No significant differences in body weight (Figure 1A) or food intake (Figure 1B) were observed among groups. Average food intake was approximately 2.5–3.0 g per mouse per day. Based on the concentration of boldine in the customized chow and measured average food intake, this corresponded to an estimated exposure of approximately 50–60 mg/kg body weight/day, compared with the nominal target of 50 mg/kg/day. Because animals were pair housed, this represents estimated average exposure rather than individually measured dosing. As expected, comparing to sham-operated animals, CCI increased the sensitivity to mechanical stimuli on the ipsilateral hindlimb at 3, 7, and 14 days post injury (dpi) (*p* < 0.001 at all three time points, Figure 1C). Oral boldine administration attenuated injury-induced mechanical allodynia at 3 dpi (*p* < 0.05) and at 7 and 14 dpi (*p* < 0.001, Figure 1C). Responses to mechanical stimuli on the contralateral hind limb did not change (Figure 1D), indicating that the effect was observed only on the injured side.

### 3.2. Boldine Did Not Alter CCI-Induced Weight-Bearing Deficits

**Figure 2:**
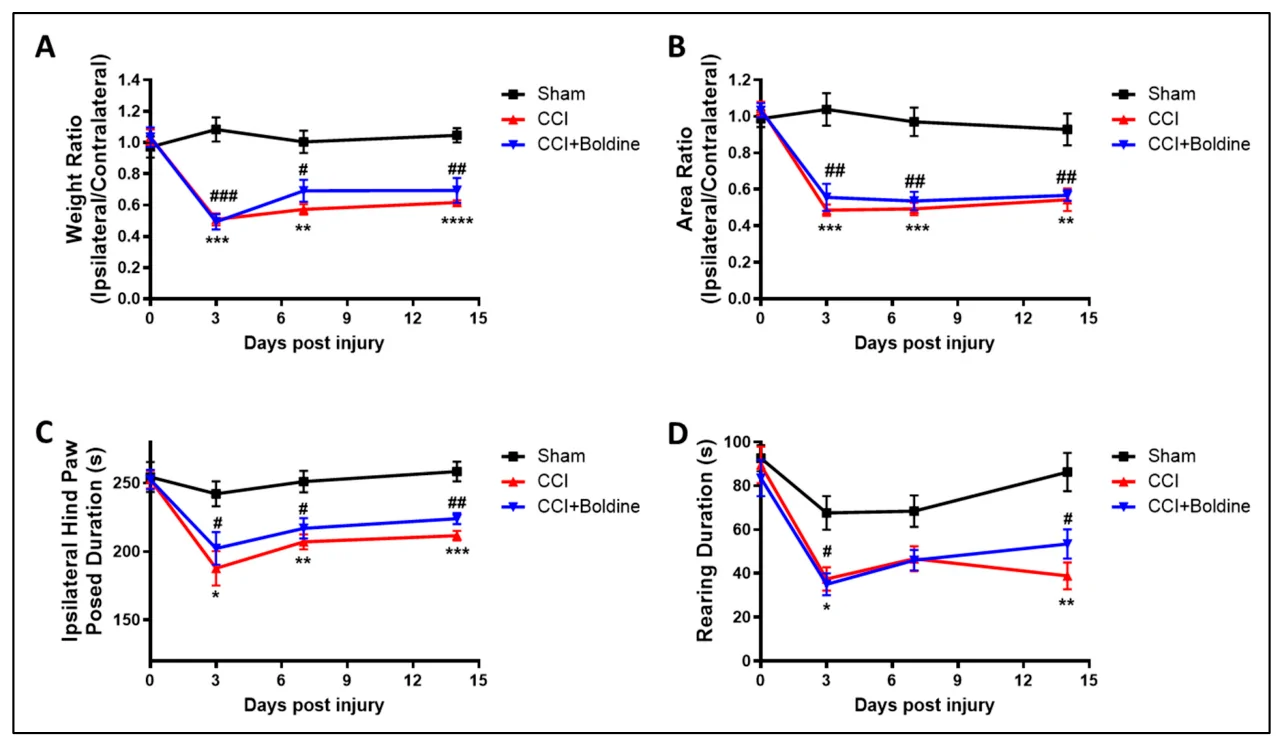
Effect of boldine on postural weight bearing phenotypes after CCI. Postural equilibrium and spontaneous weight distribution patterns were assessed via the Dynamic Weight Bearing System 2.0 (Bioseb) in sham animals (Sham) and animals with (CCI + Boldine) or without (CCI) boldine treatment. (**A**) Weight support ratio, ipsilateral hind limb vs. contralateral hind limb; (**B**) Support area ratio, ipsilateral hind limb vs. contralateral hind limb; (**C**) Ipsilateral hind paw posed duration; (**D**) Rearing duration. Data represent Mean ± SEM, N = 8 per group, * *p* < 0.05, ** *p* < 0.01, *** *p* < 0.001, **** *p* < 0.0001 (CCI vs. Sham); # *p* < 0.05, ## *p* < 0.01, ### *p* < 0.001 (CCI + Boldine vs. Sham) by two-way ANOVA with Tukey post-tests.

In CCI animal models, the reduction in weight bearing on an injured limb is a key behavioral indicator. After injury, animals demonstrate reduced weight bearing on the injured (ipsilateral) paw, which might reflect ongoing spontaneous pain [32]. To investigate the potential of boldine on modulating CCI-induced weight bearing changes, we performed weight bearing tests using the dynamic weight bearing 2.0 system, which is an automated system for quantifying postural weight-bearing phenotypes in animal models [33,34]. As compared to sham-operated mice, injured mice demonstrated significant decreased ipsilateral/contralateral weight support ratio (Figure 2A), ipsilateral/contralateral area ratio (Figure 2B), and posed duration on the ipsilateral hind limb (Figure 2C) at 3, 7, and 14 dpi. CCI also resulted in markedly lower rearing duration (*p* < 0.05 at 3 dpi, *p* < 0.0001 at 14 dpi, Figure 2D), an index of spontaneous nociceptive behavior in mice, characterized by vertical rearing where the animal supports its weight on its hind limbs while elevating its forepaws and extending its neck [35]. Although there seemed to be some trend of improvement on the parameters measured, boldine treatment did not show any significant beneficial effect on the CCI-induced weight-bearing deficits (Figure 2A–D).

### 3.3. Boldine Reduced the Expression of Inflammatory Mediators in the Spinal Cord

**Figure 3:**
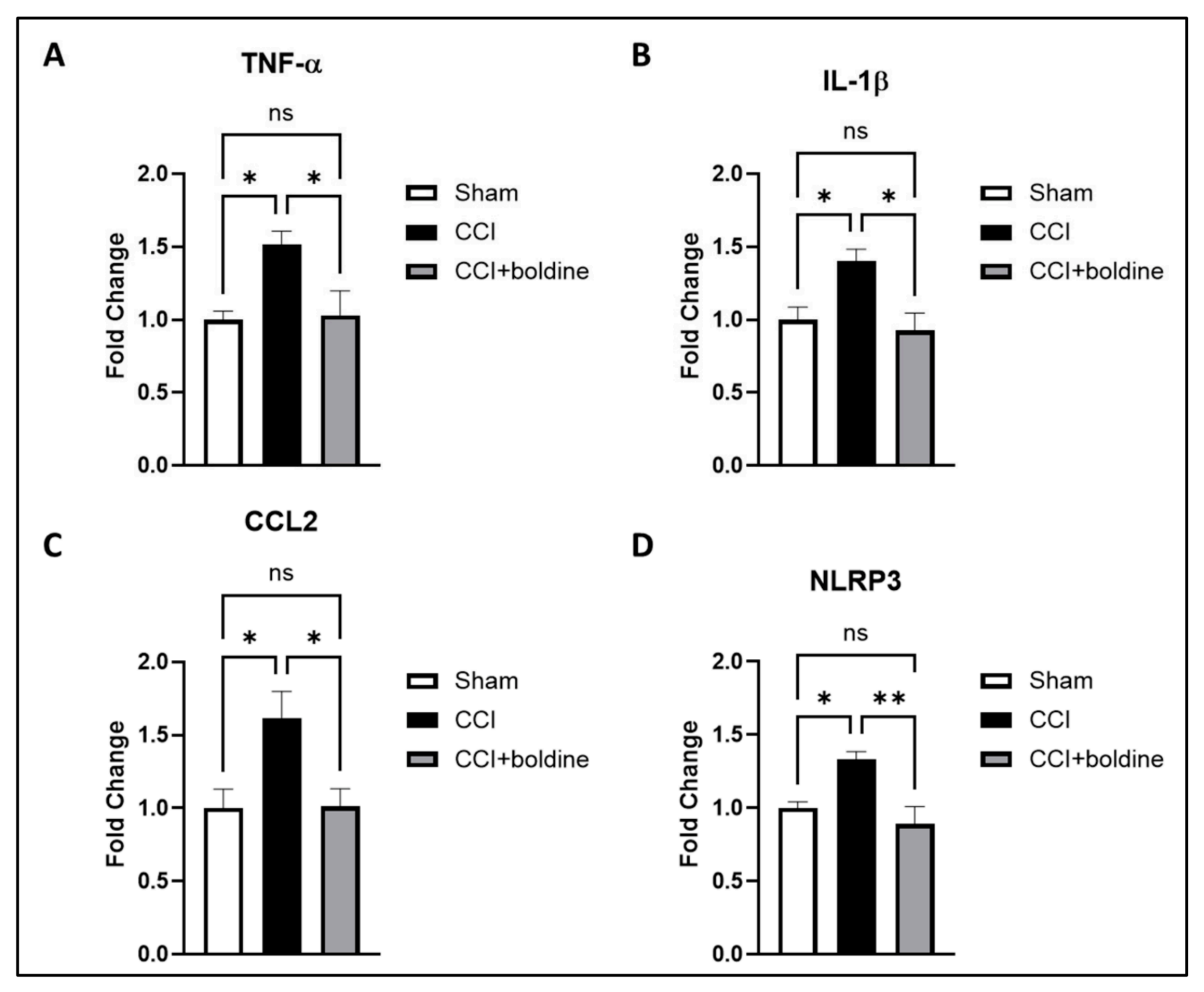
Boldine reduces CCI-induced expression of inflammatory mediators in the spinal cord. Total RNA was extracted from L3–L5 spinal cords collected at 15 dpi and subjected to gene expression analysis by quantitative PCR using standard TaqMan methods. Relative expression levels were calculated using the 2^−∆∆Ct^ method with *HPRT* as an endogenous control. (**A**) *TNF-α*; (**B**) *IL-1β*; (**C**) *CCL2*; (**D**) *NLRP3* mRNA levels. Data represent Mean ± SEM, N = 5 per group, * *p* < 0.05, ** *p* < 0.01 by one way ANOVA with Tukey posttests.

Tumor Necrosis Factor-alpha (TNF-α) and the NLRP3 (NLR family pyrin domain containing 3) inflammasome are both key players in the neuroinflammation that drives neuropathic pain, with TNF-α acting as a priming signal that upregulates *NLRP3* expression, which then activates the release of pro-inflammatory cytokines like IL-1β [36]. C-C motif chemokine ligand 2 (CCL2), also known as monocyte chemotactic protein 1 (MCP-1), plays a pivotal role in the development and maintenance of both inflammatory and neuropathic pain by promoting neuroinflammation and central sensitization [37]. To evaluate the effect of boldine on the expression of these inflammatory markers in the spinal cord following CCI, we extracted RNA from lumbar spinal cords collected at 15 dpi and performed quantitative PCR using the standard TaqMan method. Substantially elevated mRNA expression of *TNF-α*, *IL-1β*, *CCL2* and *NLRP3* was detected in the lumbar spinal cord following CCI (*p* < 0.05, Figure 3A–D). Oral administration of boldine decreased the mRNA levels of these inflammatory mediators (*p* < 0.05 for *TNF-α*, *IL-1β* and *CCL2*, Figure 3A–C; *p* < 0.01 for *NLRP3*, Figure 3D), suggesting that boldine attenuated spinal neuroinflammation by suppressing the expression of these inflammatory mediators.

### 3.4. Transcriptomic Changes in the L3–L5 Spinal Cord Following CCI and Boldine Treatment

**Figure 4:**
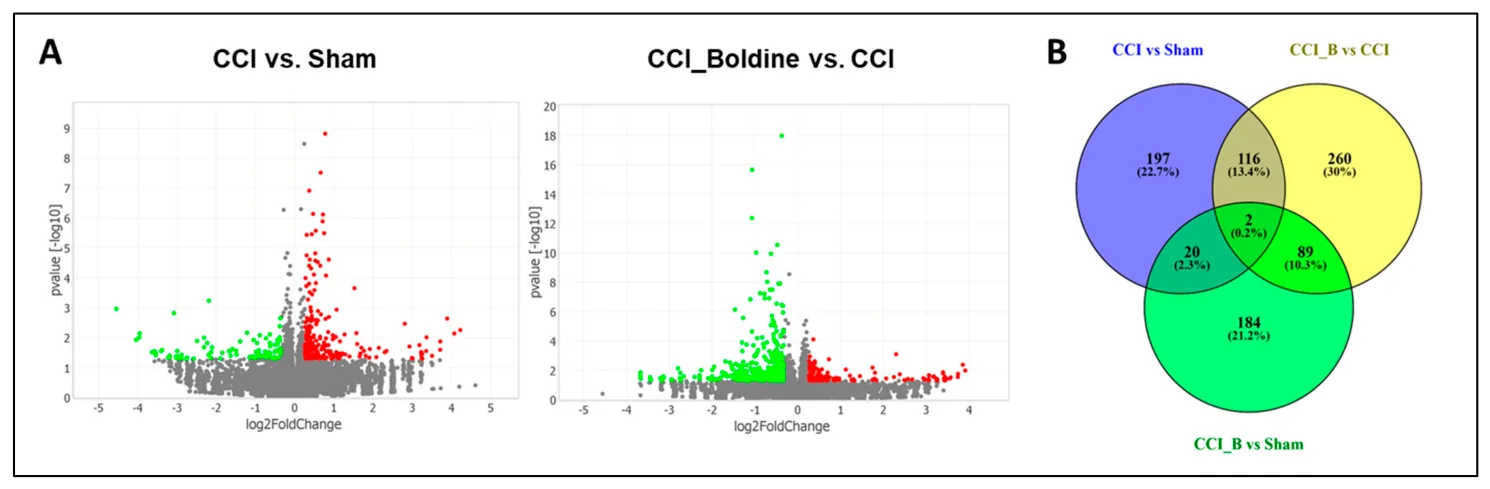
Transcriptomic changes in the spinal cord following CCI and boldine treatment. Total RNA isolated from lumbar spinal cords harvested at 15 dpi were subjected to bulk RNA sequencing and data analysis. (**A**) Volcano plots for the following comparisons: CCI vs. Sham and CCI_boldine vs. CCI, Downregulated genes are shown in green, upregulated genes are shown in red, and not significantly regulated genes are shown in grey. (**B**) Venn diagram showing the number of overlapping DEGs between comparisons.

The spinal cord serves as the initial relay point for transmitting nociceptive signals from the peripheral nervous system to the brain. We focused on the L3–L5 spinal cord to examine central molecular responses associated with CCI-induced pain processing and sensitization. This tissue selection was intended to address spinal mechanisms rather than local responses within the injured sciatic nerve or dorsal root ganglia. Bulk RNA sequencing was performed using the Illumina NextSeq 2000 platform with four independent biological replicates per group. Using the exploratory criteria of nominal *p* < 0.05 and |logFC| > log2(1.2), 335 genes (96 downregulated, 239 upregulated) met the specified criteria in CCI versus Sham, and 467 genes (335 downregulated, 132 upregulated) (Supplemental Table S1) met the specified criteria in CCI + boldine versus CCI (Figure 4A). Overlapping DEGs between comparisons were visualized using a Venn diagram (Figure 4B). These gene-level findings should be interpreted as exploratory because the reported threshold is based on nominal *p* values. Genes meeting these exploratory criteria were then subjected to Ingenuity Pathway Analysis (IPA). The top canonical pathways identified after CCI included extracellular matrix organization, GP6 signaling, integrin cell-surface interactions, and EGR2/SOX10-mediated initiation of Schwann-cell myelination (Table 1), while boldine treatment produced changes in the opposite direction for several of these pathway-level signals (Table 2).

**Figure 5:**
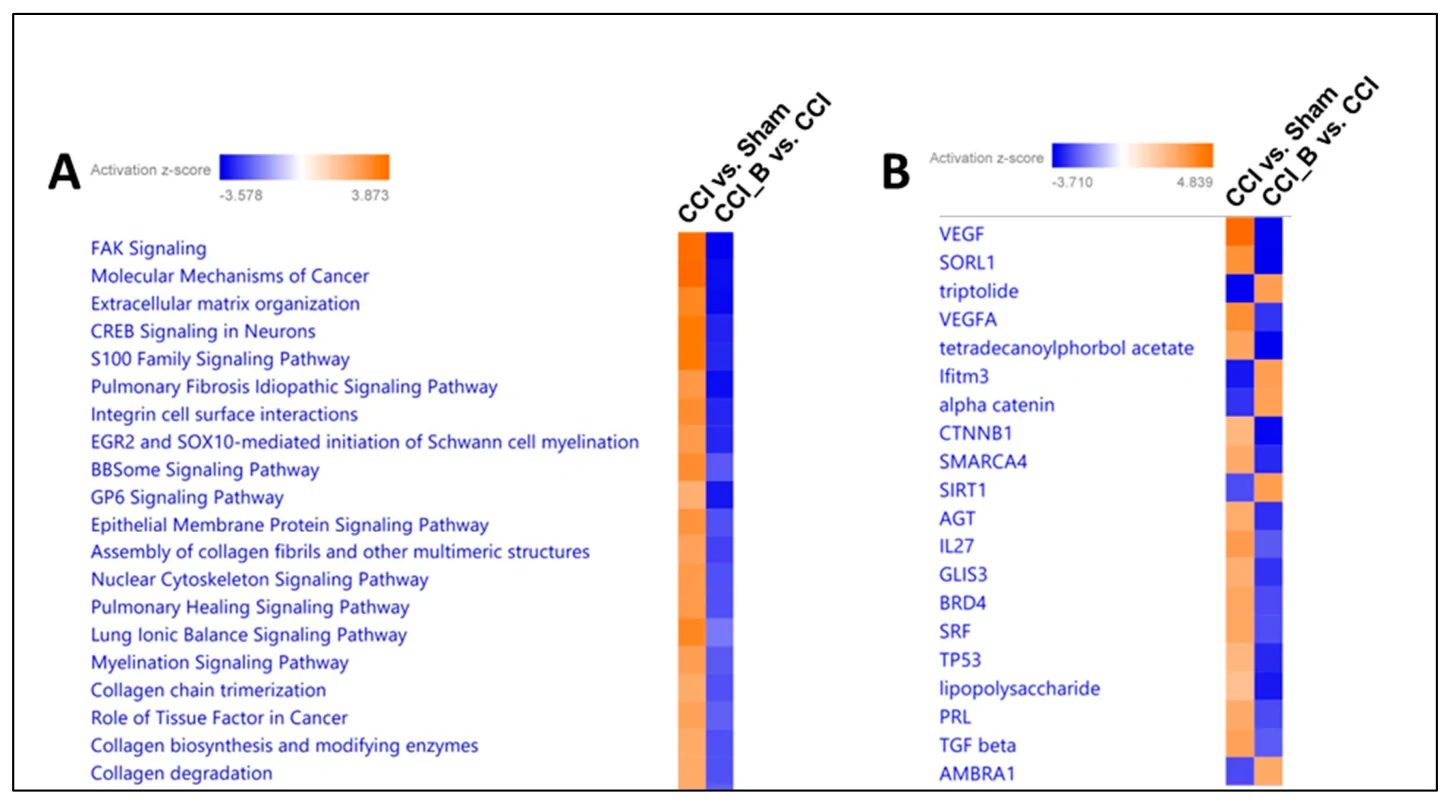
Boldine reversed CCI-induced dysregulation of canonical pathways and upstream signaling. Comparison analysis was performed using the IPA platform. (**A**) Top 20 canonical pathways identified. (**B**) Top 20 upstream factors identified. Orange indicates upregulation and blue indicates downregulation.

To understand whether boldine could reverse CCI-induced transcriptomic changes, we conducted comparison analysis in the IPA platform. The top 20 pathways that were modulated by CCI and rescued by boldine were shown in Figure 5A. Notably, several identified pathways have been implicated in the development and chronification of pain, including focal adhesion kinase (FAK) signaling, extracellular matrix (ECM) organization, CREB (cAMP response element-binding protein) signaling, and integrin cell surface interactions. Studies have shown the hyperactivation of integrin β1/FAK signaling in the spinal cord and dorsal root ganglion after nerve injury, which amplifies pain signals and contributes to chronic hypersensitivity [38]. Comprehensive transcriptomics analysis in mice and humans identified ECM organization as a central molecular pathway in the development of chronic pain [39]. Specifically, alterations in ECM components and their interactions with cells, particularly neurons and glial cells, can disrupt normal neuronal function and contribute to pain chronification. Increased CREB phosphorylation has been observed in various pain models, suggesting its involvement in both acute and chronic pain states [40]. Our data showed that the oral administration of boldine attenuated the CCI-induced activation of these pain-related pathways, suggesting potential underlying mechanisms for its analgesic effect.

Comparison analysis also revealed putative upstream factors that might be dysregulated by CCI and normalized by boldine treatment (Figure 5B). Specifically, vascular endothelial growth factor (VEGF), sortilin-related receptor 1 (SORL1), β-catenin (CTNNB1), TP53, bromodomain-containing protein 4 (Brd4), serum response factor (SRF), and transforming growth factor-beta (TGF-β) were induced by CCI and inhibited by boldine; whereas interferon-induced transmembrane protein 3 (IFITM3), α-catenin, sirtuin 1 (SIRT1) and activating molecule in beclin1-regulated autophagy 1 (AMBRA1) were downregulated by CCI and rescued by boldine. In addition, some chemical compounds were predicted to be dysregulated, including triptolide, tetradecanoylphorbol acetate (TPA) and lipopolysaccharide (LPS).

**Figure 6:**
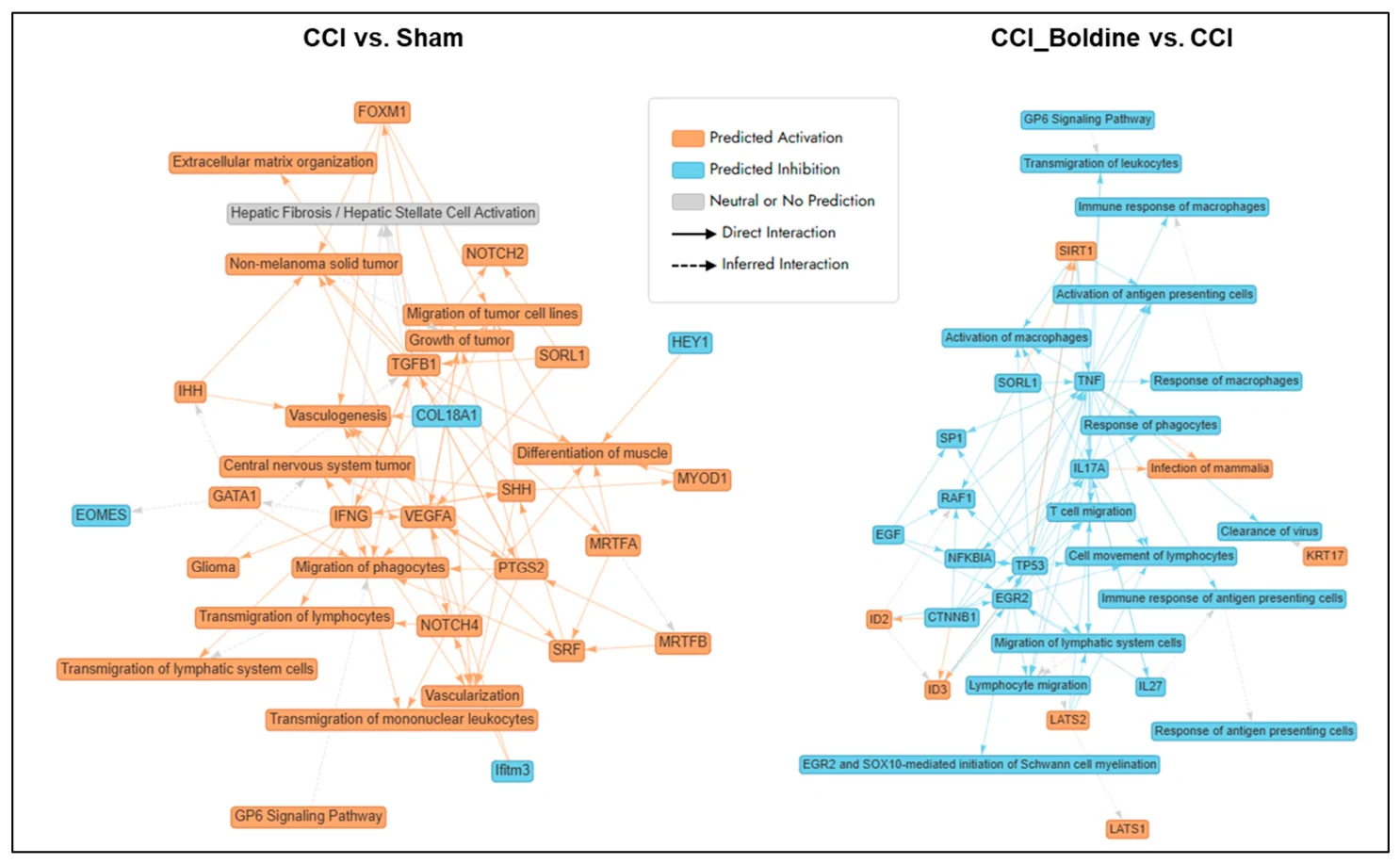
Differentially altered networks after CCI and boldine treatment. Network analysis was performed using the IPA platform. (**Left panel**) CCI vs. Sham, (**Right panel**) CCI_boldine vs. CCI. Predicted activation is shown in orange and predicted inhibition is shown in blue. Arrow shows direct interaction and dotted arrow shows indirect interaction.

Network analysis highlighted several interactions leading to the decrease of lymphocyte migration, migration of lymphatic system cells, and transmigration of leukocytes after boldine treatment (Figure 6, Right panel), suggesting a significant focus on the regulation of immune cell movement, which is crucial for immune responses, inflammation, and tissue repair mechanisms. Specifically, it revealed multiple pathways depicting the decrease in activation and immune response of antigen-presenting cells and macrophages, particularly related to IL17A, TNF-α, and TP53 activities, suggesting a predominant theme of regulating immune signaling and cytokine activity to manage response to infection and immune challenges.

## 4. Discussion

The CCI mouse model is a widely used preclinical tool to study peripheral neuropathic pain [23,24]. It mimics human neuropathic pain conditions by inducing inflammation, swelling, and localized nerve damage, which reliably leads to behavioral hypersensitivity. In this model, mechanical hypersensitivity usually manifests within 3 to 5 days, plateaus at maximum allodynia around day 7 to 14, and can last for several weeks to months. In this study, we found that boldine substantially reduced CCI-induced mechanical hypersensitivity, which is consistent with our previous findings in a mouse spared nerve injury model [21]. Changes in weight-bearing distribution are often observed as an indication of spontaneous pain-like behavior in neuropathic, inflammatory and cancer pain models [41]. We have shown that, in the SNI model, boldine attenuated injury-induced reduction of ipsilateral weight support in female mice [21]. However, no beneficial effect of boldine in correcting CCI-induced weight-bearing deficits was observed in this study. The discrepancy in correcting weight-bearing deficits we observed in these two PNI models might likely be attributed to model difference and/or sex difference. Male and female mice exhibit distinct sex differences in weight-bearing and locomotor behaviors, driven by anatomical and neurological factors. Generally, male mice tend to place more weight on their hindquarters, whereas females favor their forepaws [42]. Future studies conducted in both sexes would provide a better understanding of effect of boldine on weight-bearing behavior and the underlying mechanisms.

After PNI, neuroinflammation acts as the primary driver of central sensitization. Glial cell activation drives neuroinflammation in the brain and spinal cord, where reactive microglia and astrocytes release pro-inflammatory chemokines and cytokines [43]. In this study, boldine attenuated CCI-associated increases in spinal inflammatory transcripts, and exploratory bulk RNA sequencing of the L3–L5 spinal cord identified pathway-level changes involving inflammation and immune responses. These molecular findings are consistent with an anti-inflammatory effect but do not by themselves establish a causal mechanism or structural neuroprotection. Several limitations should therefore be considered. First, boldine treatment was initiated immediately after CCI, so the study evaluates early intervention and cannot determine whether boldine reverses established neuropathic pain. Second, molecular analyses were limited to the L3–L5 spinal cord; the injured sciatic nerve and dorsal root ganglia were not examined, and the sampled spinal region does not encompass the full L4-S3 distribution associated with sciatic input. Third, histological and immunohistochemical analyses were not performed, so the present study cannot determine whether boldine prevents structural nerve damage, preserves myelin, promotes axonal regeneration, or alters the morphology of glial or immune-cell activation. Finally, the RNA-seq analysis included only four biological replicates per group and should be interpreted as exploratory and hypothesis generating. The limited sample size reduces statistical power for gene-level inference and is considered when interpreting the transcriptomic findings. Future studies incorporating delayed treatment, analysis of DRG and sciatic nerve, histological endpoints, both sexes, and larger transcriptomic cohorts will be important to establish mechanism and reproducibility.

Upstream analysis of spinal cord transcriptomic data revealed several putative upstream factors that have been indicated in pain development. For example, VEGF sensitizes sensory nerves and promotes inflammatory processes in chronic conditions like osteoarthritis and neuropathic pain [44]. Upregulation of β-catenin signaling has been shown as a common pathway leading to various phenotypes of spinal degeneration and pain [45]. BRD4 has been implicated in inducing chronic postsurgical pain through activating TLR4/NF-κB-dependent neuroinflammation [46], and inhibiting BRD4 can alleviate inflammatory pain by inhibiting NLRP3 inflammasome [47]. SRF induces hyperalgesia-priming behavior and may contribute to the chronification of pain [48]. SIRT1 expression is reduced in the spinal cord following nerve injury, and spinal SIRT1 activation attenuates neuropathic pain in mice [49,50]. Our data showed that oral administration of boldine not only decreased the elevation of pain-causing factors but also restored the levels of pain-suppressing factors, which is consistent with our observed analgesic effect. Several chemicals were also identified as upstream factors. Specifically, triptolide has been shown to reduce neuropathic pain by inhibiting immune responses [51,52], and TPA and LPS are inflammatory-inducing agents. Boldine treatment not only reversed the downregulation of triptolide signaling but also reversed the upregulation of TPA and LPS signaling, suggesting an anti-inflammatory mechanism following CCI. Interestingly, key genes involved in oncogenesis such as TP53 and TNF were depicted as central nodes in the regulated network. Their decrease by boldine treatment correlated with changes in immune responses and cellular migration, indicating themes of tumor suppression, cancer development, and the immune system’s role in these processes.

In summary, early oral administration of boldine attenuated CCI-induced mechanical allodynia and was associated with reversal of multiple injury-related transcriptional changes and reduced expression of inflammatory mediators in the spinal cord of male mice. These findings provide preliminary preclinical evidence that boldine can modulate molecular and behavioral responses following peripheral nerve injury and support further investigation of its mechanisms and therapeutic potential. Future studies incorporating both sexes, delayed-treatment paradigms, additional peripheral and central nervous system tissues, histological analyses, and larger confirmatory cohorts will be required to determine its efficacy in established neuropathic pain and its translational relevance.

## Figures and Tables

**Table 1:** Top 10 canonical pathways (CCI vs. Sham).

Pathway	*p*-Value	Activation z-Score	Percentage Overlap
EGR2 and SOX10-mediated initiation of Schwann cell myelination	3.5 × 10^−9^	2.65	24.14%
Hepatic Fibrosis/Hepatic Stellate Cell Activation	9.6 × 10^−9^	-	6.77%
Extracellular matrix organization	2.4 × 10^−8^	3.16	9.35%
Integrin cell surface interactions	4.1 × 10^−8^	3	10.59%
Assembly of collagen fibrils and other multimeric structures	1.2 × 10^−5^	2.45	9.84%
GP6 Signaling Pathway	1.3 × 10^−5^	2.12	6.25%
Collagen chain trimerization	3.3 × 10^−5^	2.24	11.36%
Dilated Cardiomyopathy Signaling Pathway	4.3 × 10^−5^	1.63	5.30%
Pulmonary Fibrosis Idiopathic Signaling Pathway	1.0 × 10^−4^	2.71	3.37%
Phagosome Formation	1.0 × 10^−4^	2.18	2.41%

**Table 2:** Top 10 canonical pathways (CCI_boldine vs. CCI).

Pathway	*p*-Value	Activation z-Score	Percentage Overlap
Extracellular matrix organization	5.6 × 10^−13^	−3.36	14.02%
EGR2 and SOX10-mediated initiation of Schwann cell myelination	1.3 × 10^−11^	−3	31.03%
tRNA processing in the mitochondrion	1.1 × 10^−8^	−2.12	19.51%
rRNA processing	3.9 × 10^−8^	−2.65	21.88%
lntegrin cell surface interactions	3.2 × 10^−7^	−3	10.59%
Sertoli Cell-Sertoli Cell Junction Signaling	4.8 × 10^−7^	−1.07	5.67%
GP6 Signaling Pathway	1.2 × 10^−6^	−3.16	7.81%
Myelination Signaling Pathway	2.8 × 10^−6^	2.32	4.57%
Wound Healing Signaling Pathway	3.1 × 10^−6^	−1.94	5.22%
Assembly of collagen fibrils and other multimeric structures	3.9 × 10^−6^	−2.65	11.48%

## Data Availability

The datasets generated and analyzed during the current study are available in the GEO repository under accession number GSE344718.

## Supplementary Materials

The following supporting information can be downloaded at: https://www.mdpi.com/article/10.3390/biomedicines14102345/s1. Table S1: List of Differentially Regulated Genes Between Groups.

## Abbreviations

The following abbreviations are used in this manuscript:

ANOVA	Analysis of Variance
BRD4	Bromodomain-containing protein 4
BW	Body weight
CCI	Chronic constriction injury
CCL	C-C motif chemokine ligand
CREB	cAMP response element-binding protein
CTNNB1	β-catenin
DEG	Differentially expressed gene
DWB	Dynamic weight bearing
ECM	Extracellular matrix
FAK	Focal adhesion kinase
HPRT	Hypoxanthine phosphoribosyltransferase
IL	Interleukin
LPS	Lipopolysaccharide
MCP1	monocyte chemotactic protein 1
NF-κB	Nuclear factor kappa-light-chain-enhancer of activated B cells
NLRP	Nucleotide-binding oligomerization domain, Leucine-rich repeat, and Pyrin domain containing
PNI	Peripheral nerve injury
SEM	Standard error of the mean
SNI	Spared nerve injury
SORL1	Sortilin-related receptor 1
TGF-β	Transforming growth factor-beta
TLR	Toll-like receptor
TNF	Tumor necrosis factor
TPA	Tetradecanoylphorbol acetate
VEGF	Vascular endothelial growth factor
